# Attachment Avoidance Is Significantly Related to Attentional Preference for Infant Faces: Evidence from Eye Movement Data

**DOI:** 10.3389/fpsyg.2017.00085

**Published:** 2017-01-26

**Authors:** Yuncheng Jia, Gang Cheng, Dajun Zhang, Na Ta, Mu Xia, Fangyuan Ding

**Affiliations:** ^1^School of Psychology, Southwest UniversityChongqing, China; ^2^Center for Mental Health Education, Southwest UniversityChongqing, China; ^3^School of Educational Science, Guizhou Normal UniversityGuiyang, China; ^4^School of Educational Science, Guangxi University for NationalitiesNanning, China

**Keywords:** adult attachment, parenting, infant face, eye-tracking, visual attentional bias

## Abstract

**Objective:** To determine the influence of adult attachment orientations on infant preference.

**Methods:** We adopted eye-tracking technology to monitor childless college women’s eye movements when looking at pairs of faces, including one adult face (man or woman) and one infant face, with three different expressions (happy, sadness, and neutral). The participants (*N* = 150; 84% Han ethnicity) were aged 18–29 years (*M* = 19.22, *SD* = 1.72). A random intercepts multilevel linear regression analysis was used to assess the unique contribution of attachment avoidance, determined using the Experiences in Close Relationships scale, to preference for infant faces.

**Results:** Women with higher attachment avoidance showed less infant preference, as shown by less sustained overt attentional bias to the infant face than the adult face based on fixation time and count.

**Conclusion:** Adult attachment might be related to infant preference according to eye movement indices. Women with higher attachment avoidance may lack attentional preference for infant faces. The findings may aid the treatment and remediation of the interactions between children and mothers with insecure attachment.

## Introduction

Attachment theory (Bowlby, 1969) has become a major theoretical perspective used in the study of relationships. Attachment is considered a significant psychological system that guides thoughts, feelings, and behaviors in relationships across the lifespan (Bowlby, 1979). As a dynamically interactive system, attachment is perhaps most critical during the early stages of life; however, Bowlby (1988) assumed that this system is active over the entire lifespan, thereby providing a solid theoretical foundation for understanding and studying the links between adult attachment and parenting. Many subsequent studies have drawn from Bowlby’s theory for that purpose.

There are two interactional behavioral systems—attachment and caregiving—at work in Bowlby’s attachment theory, and they may not always be perfectly balanced (Bowlby, 1969). For example, increased activation of the parent’s attachment system may reduce the parent’s caregiving system activation (Bowlby, 1969). Under such circumstances, the parent’s attachment-related thoughts, needs, and coping strategies may interfere with their ability to respond appropriately to the child’s needs. Women, as primary caregivers (Bretherton et al., 2006), are more sensitive to infants’ needs and are more involved in infant caretaking activities (Dulac et al., 2014). As such, mothers’ attachment styles have a significant influence on the formation and development of parenthood and, thereby, the mental and physical health of their offspring, which includes the infant’s survival and development of cognitive, emotional, and socialization skills (Sroufe, 2005; Dulac et al., 2014). Thus, research into attachment and parenting has direct relevance for improving the quality of nurturing provided to the infant.

Infants possess certain characteristics that are mainly concentrated in the face and are often conceptualized as the “baby schema” (Lorenz, 1943). The preference for infant faces (hereafter, “infant preference”), which is embodied in attentional preference, positive emotion/attitude and physiological reward, is an important research topic in the association between attachment and parenting. First, researchers found attentional bias toward infant faces compared to adult faces (Brosch et al., 2007), and this preference showed consistency across cultures (Cárdenas et al., 2013; Charles et al., 2013). Moreover, the more obvious infant schema the baby faces, the longer the gaze time (Power et al., 1982). Second, baby schema can make people want to care for the baby, respond to its needs in a timely manner, and be more willing to expend effort in caring for it (Brosch et al., 2007, 2008). Moreover, the more obvious the baby schema, the more cute the baby faces, the more likely to be judged friendly, healthier and competitive (Ritter et al., 1991). Third, some studies have shown that infant faces are uniquely attractive and hedonic stimuli (Lorenz, 1971; Gould, 1980), effectively activating reward related areas in the brain (Strathearn et al., 2008; Glocker et al., 2009), that are consistently preferred by children, sexually mature adolescents, and adults (Berman et al., 1975; Fullard and Reiling, 1976; Sanefuji et al., 2007). All of these efforts are conducive to the growth of the infant and enable ethnic groups to adapt and develop, thereby promoting the survival of the human race.

Many researchers have explored the relationship between attachment in adulthood (e.g., self-reported and interview studies) and various facets of parenting. In general, the results have consistently shown that across three aspects of parenting (i.e., cognition, emotion, and behavior), insecure attachment is negatively related to parenting characteristics and outcomes (Jones et al., 2015). Many studies have indicated that adult attachment is related to infant preference. Specifically, both avoidant and anxious mothers had higher expectations of being aggravated by their children (Kwako et al., 2010; Trillingsgaard et al., 2011). The anxiety levels of mothers was positively correlated with the degree of hostile feelings toward the infant (Scher and Dror, 2003). Moreover, avoidant college women and couples had less desire to have children and garnered less satisfaction from caring for infants (Rholes et al., 1997, 2006). Both an avoidance and anxiety attachment in mothers was negatively correlated with parental self-efficacy (Kohlhoff and Barnett, 2013) and related to perceptions of infant temperament as being more fearful, more negatively reactive, and less adaptable (Pesonen et al., 2003).

Following the behavior paradigms used by Heerey and Gold (2007), researchers discovered that when viewing images of infant faces, compared to adult faces, the higher security attachment state was associated with higher levels of self-reported interest in infants, and associated with liking and wanting (divided into representational and evoked responding) for infants’ neutral faces, showing positive motivational behaviors; moreover, avoidance attachment state was positively related to infant faces elicited pleasure (Cheng et al., 2015a).

With the development of cognitive neuroscience and neurobiology, researchers have begun studying the physiological mechanisms underlying attachment and infant preference, using functional magnetic resonance imaging (fMRI). For example, differences in adult attachment correlated to maternal brain and oxytocin response to infant cues (Strathearn et al., 2009). In this study, mothers with secure attachment who viewed their own infant’s smiling and crying faces showed increased activation of the mesocorticolimbic regions (i.e., the reward brain), whereas mothers with insecure attachment showed greater activation of the anterior insular region, which is associated with feelings of unfairness, pain, and disgust, in response to their own infant’s sad facial expression (Montague and Lohrenz, 2007).

Although the above studies, adopting various methods (e.g., self-reported, behavior and fMRI), may suggest that adult attachment is related to infant preference, this relationship is still not very well understood. One point that has not yet been considered in this relationship is the fact that, because newborn babies do not yet have language, parents and children use eye contact as an important communication channel. Research investigating how eye movements reflect infant preference may contribute valuable information to our understanding of parental bonding. Indeed, many studies have shown that infants have strong preferences for their mother’s face (Brazelton et al., 1975; Blehar et al., 1977; Field, 1977). Furthermore, from around 4 months of age, human infants show preferences for viewing faces (Farroni et al., 2002), which is considered to reflect a vital stage in subsequent social development (Baron-Cohen et al., 1985). Thus, the use of eye-tracking technology may supplement the evidence obtained from self-reports and brain imaging technologies by providing an overt behavioral perspective.

Eye-tracking technology is likely to have high ecological validity. By allowing continuous recording of attention, eye tracking provides an important advancement in characterizing the time course and components of attentional bias (Armstrong and Olatunji, 2012). For example, the initial orienting of overt attention to a stimulus can be distinguished easily from subsequent dwell time (DT), as orienting is reflected in saccade sequences (i.e., where one looks), whereas DT is reflected in fixation durations (i.e., how long one looks). Widely used eye indicators include the fixation DT and the fixation counts (FC) on the area of interest. DT (i.e., longer looks), which is total amount of fixation durations on the area of interest, is the best index to compare the distribution of attention on different stimuli. Moreover, the more FC (i.e., more looks), the more important the area for the observer (i.e., captured more attention). Therefore, when different stimuli are presented at the same time, eye-tracking technology can not only be used to study early stages of attention, but also can accurately describe the characteristics of sustained attention under conscious control. This enhances the shortcomings of simple behavioral experiments that explore attentional bias, and provides more objective indices to describe the behavioral response to infant schema.

On the other hand, it is necessary to explore the influences of varying facial emotions. First, the functional neuroanatomy of caregiving and that the anterior cingulate may play an important role in thinking about emotional stimuli such as infant faces and shifting attention toward such stimuli (Swain, 2011). Second, infants’ smiling or crying facial expressions convey their emotional state (Fridlund, 1997) and need (Trivers, 1974); however, most studies only used neutral infant faces as stimuli (Yamamoto et al., 2009; Parsons et al., 2011; Charles et al., 2013; Cheng et al., 2015a). Third, when an individual scores high on dimensions of anxious or avoidant attachment, they are more likely than those who are securely attached to avoid looking at attachment-related threatening stimuli (Dewitte et al., 2007; Dewitte and De Houwer, 2008). Lastly, avoidant individuals are more likely to avoid overtly attending to emotional information (e.g., both happy and angry faces) in general, rather than angry faces specifically (Byrow et al., 2016).

Based on the above background, this study examined how individual differences in adult attachment relate to infant preference using eye-tracking technology. We proposed that women with high scores on dimensions of anxious and avoidant attachment would negatively correlate with infant preference, they would show less sustained overt attentional bias to infant faces compared to adult faces, and that attentional bias will vary according to various facial expressions.

## Materials and Methods

### Participants

We recruited 150 women at our university. The participants were unmarried, childless, and 18–29 years old (*M* = 19.22, *SD* = 1.72). The majority (over 84%) of the sample was of Han ethnicity. All reported having normal or corrected-to-normal vision. One participant looked around, rather than at, either of the faces on many of the trials, as evidenced by her eye movement data, so she was not included in the final analysis. Participation was anonymous, and the participants were compensated with 15 RMB. The ethics committee of our university (No. 2014179) approved this study.

### Experiences in Close Relationships (ECR)

**FIGURE 1:**
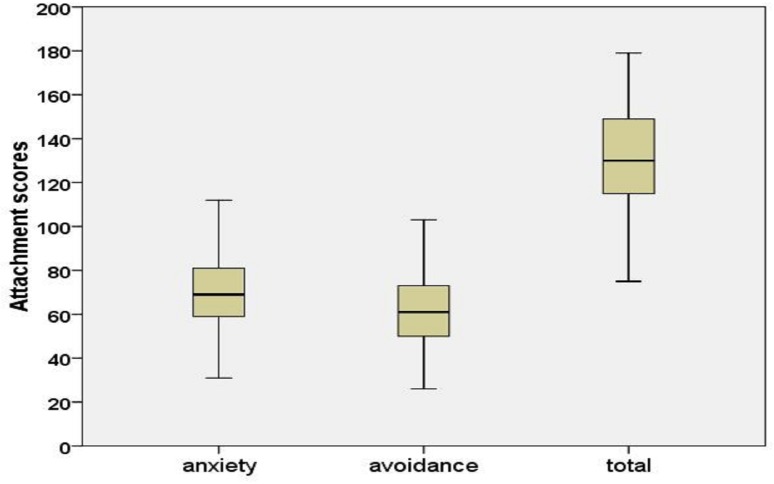
**Attachment items distribution**.

The Experiences in Close Relationships (ECR; Brennan et al., 1998) is the most commonly used measure of general patterns of adult attachment. It contains 18 items that assess attachment anxiety and 18 items that assess attachment avoidance (36 items total). Items are rated on a 7-point Likert-type scale, ranging from one (strongly disagree) to seven (strongly agree), with four (neutral/mixed) being the midpoint of the scale. People who score high on either or both of these dimensions are assumed to have an insecure adult attachment orientation. In contrast, people with low levels of attachment anxiety and avoidance can be viewed as having a secure adult attachment orientation (Brennan et al., 1998; Lopez and Brennan, 2000; Mallinckrodt, 2000). In the present study, we employed the Chinese version of the ECR, which was translated and revised by Li and Kato (2006). It has been shown to have well-established psychometric properties, including adequate internal consistency reliability (i.e., a Cronbach’s α of 0.82 for the avoidance subscale and 0.77 for the anxiety subscale). In the present study, the Cronbach’s α values of the avoidance and anxiety subscales were 0.82 and 0.87, respectively. Given the D’Agostino–Pearson Test (Dagostino and Pearson, 1973), the hypothesis of normality would be rejected at the 0.05 level if the test statistic *DP* = *Zs^2^* + *Z_k_^2^* is bigger than about 6 (avoidance: *DP* = 1.131; anxiety: *DP* = 0.293; total: *DP* = 2.201). The ECR roughly followed a normal distribution (see **Figure 1**): Skewness *Cs* (avoidance: *C_s_* = 0.211, *SE* = 0.199, *Zs* = *C_s_*/*SE* = 1.062; anxiety: *C_s_* = 0.105, *SE* = 0.199, *Zs* = *C_s_*/*SE* = 0.527; total: *C_s_* = -0.134, *SE* = 0.199, *Zs* = *C_s_*/*SE* = -0.676) and Kurtosis *C_k_* (avoidance: *C_k_* = -0.023, *SE* = 0.395, *Z_k_* = *C_k_*/*SE* = -0.059; anxiety: *C_k_* = 0.050, *SE* = 0.395, *Z_k_* = *C_k_*/*SE* = 0.126; total: *C_k_* = -0.521, *SE* = 0.395, *Z_k_* = *C_k_*/*SE* = -1.321). A confirmatory factor analysis (CFA) was conducted for the ECR, using a maximum likelihood estimation method in Mplus 7.4 (Muthén and Muthén, 2012). The fit indices of CFA for the ECR have an interval with different values: the worst fit (using original items): χ^2^ (593) = 2387.09, *p <* 0.0001; CFI = 0.728; TLI = 0.744; RMSEA = 0.086 [90% CI = 0.083, 0.090]; SRMR = 0.097 (Pedersen et al., 2015) and the best fit (using item parcels): χ^2^(8) = 18.25; CFI = 1.00; RMSEA = 0.03; GFI = 0.99; AGFI = 0.99; NNFI = 1.00 (Alonso-Arbiol et al., 2008). The current avoidance-anxiety two-factor model (using original items) provided a reasonable fit for the data as follows: χ^2^(541) = 854.523, *p <* 0.0001; CFI = 0.877; TLI = 0.856; RMSEA = 0.062 [90% CI = 0.054, 0.070]; SRMR = 0.096. Due to CFA based on the original items, fit indices of current model are not good, as the similar to previous studies (e.g., Pedersen et al., 2015). However, by using item parcels, fit indices of CFA are good (e.g., Alonso-Arbiol et al., 2008). Following the item parcels procedure used by Alonso-Arbiol et al. (2008), our model provided a good fit for the data as follows: χ^2^(8) = 9.31; CFI = 0.995; TLI = 0.991; RMSEA = 0.033; SRMR = 0.031.

### Stimuli

The stimuli used for eye tracking were 144 front-view images of faces with happy, sad, or neutral expressions (72 young adults and 72 infants). Each expression included 24 infants, 12 adult men, and 12 adult woman images. By independent sample *t*-tests, there were no significant differences between the infant images and adult images in the intensity of each facial expression [happy: *t*(46) = 0.301, *p* = 0.77; sad: *t*(46) = 0.214, *p* = 0.83; and neutral: *t*(46) = 0.755, *p* = 0.45]. These images of faces, including the index of facial expression intensity, were respectively taken from the Chinese Affective Face Picture System (Gong et al., 2011) and the Chinese Infant Affective Face Picture System (Cheng et al., 2015b). All faces were standardized to look straight ahead and were clipped to show only the face outline (the hair, ears, and background were removed). Any non-face area contained within the image (260 × 300 pixels) was filled in with a black background (RGB: 0, 0, 0). All images were presented in grayscale and were matched for size and luminosity through standardized batch progressing in MATLAB 7.1 (The Mathworks, 1993) using the SHINE toolbox plug-in (Willenbockel et al., 2010).

### Apparatus

The experimental task was programmed using E-prime stimulus presentation software (Psychology Software Tools, Inc., Pittsburgh, PA, USA). The faces were presented on a 19.7-inch CRT monitor. The display resolution was set to 1024 × 768 pixels, with a refresh rate of 85 Hz. Participants were asked to put their head on a chin strap set such that the distance between the eyes and the screen was 70 cm. Participants’ eye movements were monocularly recorded (right eye, Pupil-CR tracking mode) at a sampling rate of 250 Hz using the eye-tracking system EyeLink 1000 Plus Desktop Mount (SR Research Ltd., Mississauga, ON, Canada).

### Procedure

We adopted the eye-tracking paradigm employed by Cárdenas et al. (2013) to monitor participants’ eye movements while they looked at pairs of adult and infant faces with the same facial expression: happy, sadness, or neutral. Participants were first introduced to the experimental requirements, and then they were asked to sign the informed consent form. Next, they completed a demographics questionnaire and the ECR. After completing the questionnaire, they participated in the eye-tracking experiment. The whole process took about 35–40 min.

Before starting the experimental trials, the eye tracker was calibrated with the participant, and the eye positions were validated. For the calibration, the participants were asked to track 9 random points on the display; the eye tracker was then adjusted until the average tracking error of the visual angle was less than 0.4°. The validation procedure measured the difference between the computed fixation position and the fixation position for the target obtained during calibration using the same 9 random points. The experimental trials began after the eye positions were validated. The drift correction was executed before the beginning of each trial; ensuring participants always began fixated on the cross at the center of the screen. If accuracy was low, the eye tracker was recalibrated.

There were two blocks in the experimental trial (midway through, the participants had a 1-min rest period). In the first block, 72 pairs of faces were shown (24 for each of the three facial expressions), each consisting of one adult face (man or woman) and one infant face (boy or girl) and with the same facial expressions. For each pair, one face (260 × 300 pixels) was shown above the center (*x*: 512, *y*: 576), and the other was below (*x*: 512, *y*: 192). Vertical placements were chosen to minimize the laterality salience of emotional valence in the left visual field, suggesting a right hemisphere advantage (Borod et al., 2001; Brosch et al., 2007). Each face pair was presented for 6 s. Each participant received an exclusive set of randomly paired infant and adult faces through the counterbalancing of display location (above or below center), age (adult or infant), and gender (male or female) across the 144 trials.

**FIGURE 2:**
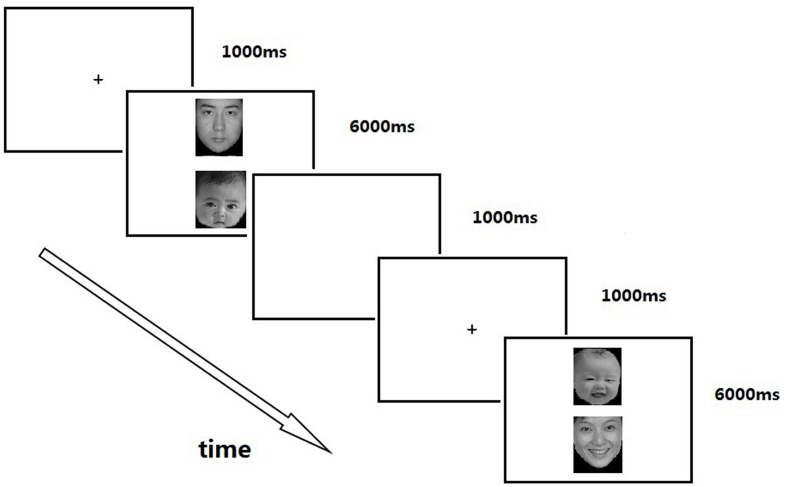
**Trial sequence**.

The experimental procedure in every trial was as follows (see **Figure 2**). A fixation cross was shown at the center of the screen for 1000 ms, and then it disappeared. Next, a pair of pictures was presented vertically for 6000 ms. The interval of each trial was 1000 ms, after which the sequence was repeated. Participants were instructed to fixate on the cross and look at the pictures in any way they wanted to.

### Data Analysis

Each face image was defined as an area of interest. Following Werthmann et al. (2011), fixations were defined as any period that was not a blink or saccade (with default thresholds automatically done by the eyetracking system) and lasted at least 100 ms (Eyelink Dataviewer User’s Manual, 2002–2008, SR Research Ltd.). Outside the area of interest, the fixation points were excluded from analysis. Three eye movement indices of attentional bias were considered: location of first fixation (FF), total amount of fixation dwell time on the area of interest for each face (DT), and total number of fixations on the area of interest for each face (FC). Eye movements were extracted using Data Viewer (SR Research Ltd., Missisauga, ON, Canada). There were dropouts of eye-tracking data due to poor eye-tracking quality (3.4%) in 144 trials. In the present study, we used visual attentional bias to adult faces as the baseline; in other words, a difference score was calculated to represent infant bias via attentional bias (i.e., the three eye movement indices). Thus, attentional bias to adult faces was subtracted from the attentional bias to infant faces, such that positive scores indicated more infant bias and negative scores indicated more adult bias.

Finally, a random intercept multilevel linear regression was used to examine the unique contribution of adult attachment in predicting preferences for the infants. Facial expressions were operationalized through virtual variable coding and were entered in the first level, while adult attachment was entered in the second level.

## Results

### Preliminary Analyses

**FIGURE 3:**
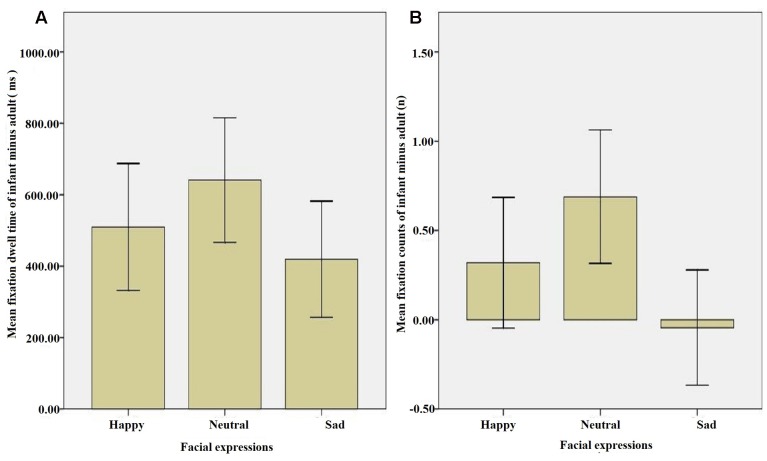
**The means and standard deviations of eye movement indices.** Error bars represent the standard error of mean. **(A)** Fixation dwell time = total amount of fixation dwell time on the area of interest for each face. **(B)** Fixation counts = total number of fixations on the area of interest for each face.

**FIGURE 4:**
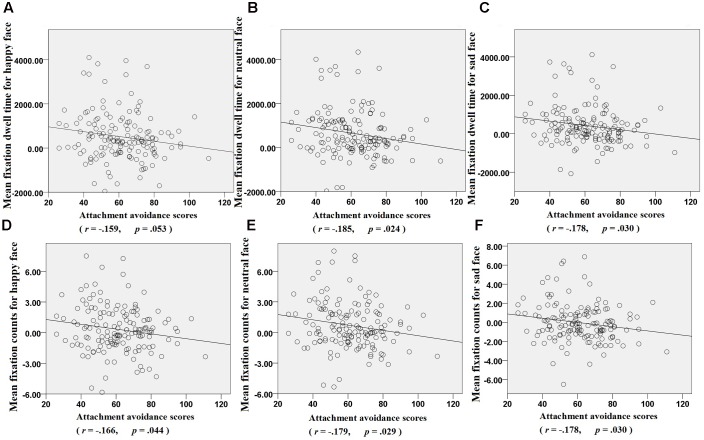
**Correlations between fixations and attachment avoidance scores**. **(A–C)** Fixation dwell time = total amount of fixation dwell time on the area of interest for each face. **(D–F)** Fixation counts = total number of fixations on the area of interest for each face.

Pearson’s bivariate correlations and descriptive statistics are presented in **Table 1**. Comparisons of the mean scores (see **Figure 3**) indicated that the infant face scores were higher than those of the adult faces for the happy and neutral facial expressions for both DT and FC, and for the sad DT facial expression. Furthermore, the difference scores (i.e., the three expressions of the infant minus those of the adult) for DT and FC were significantly negatively associated with adult attachment avoidance (see **Figure 4**), thus supporting the current hypothesis. Attachment anxiety was not significantly associated with the difference scores; therefore, no further analyses were performed to examine it.

**Table 1:** Descriptive statistics and correlations.

	Avoidant	Anxious	*M*	*SD*
Avoidant			61.228	15.972
Anxious	-0.087	-	69.565	17.577
DT-Happy	-0.159*	0.014	510.032	1097.597
DT-Neutral	-0.185*	-0.017	641.342	1076.583
DT-Sad	-0.178*	-0.028	419.342	1006.206
FC-Happy	-0.166*	0.007	0.319	2.259
FC-Neutral	-0.179*	0.010	0.689	2.310
FC-Sad	-0.178*	-0.025	-0.044	1.994
FF-Happy	0.072	-0.054	-2.409	5.094
FF-Neutral	0.112	-0.072	-0.792	4.353
FF-Sad	-0.054	-0.136	-1.644	4.640


*DT, fixation dwell time; FC, fixation counts; FF, first fixation; Happy, happy facial expression of infant minus adult; Neutral, neutral facial expression of infant minus adult; Sad, sad facial expression of infant minus adult. ^∗^*p* < 0.05.*

**Table 1** also shows that the location of FF did not significantly correlate with attachment avoidance or anxiety. Given this lack of effect of FF (i.e., locating the cross at the center of display; Cárdenas et al., 2013), it was not included in any further analyses.

### Multilevel Regression

#### Fixation Dwell Time for Infant Faces

For DT, a positive score meant longer viewing of infant faces, whereas a negative score meant longer viewing of adult faces. We analyzed infant attentional bias scores by random intercept multilevel linear regression, with facial expressions (for Level 1, in which DT was the dependent variable) nested within individual adult attachment (for Level 2, in which the intercept and slope parameters from the previous level were the dependent variables). Models were estimated with HLM (Hierarchical Linear and Non-linear Modeling) software (version 6.06) and were compared using likelihood ratio tests.

As shown in **Table 2**, the estimated value of the neutral faces, as a baseline, was significant at 641.342. This indicated that the DT bias was influenced by the neutral infants’ faces. The sad estimated value, which was also significant, was the result of subtracting 222 from 641.342. The happy estimate was similar to that of the sad. The “avoidant” estimated value, as the second-level baseline, was statistically significant, suggesting that attachment avoidance could weaken the effect of the neutral face expressions on the DT bias. Particular attention should be paid to the sad × avoidant estimate (-12.471 plus 1.282), which was not significant. The happy × avoidant estimate had a similar result. Therefore, the individual-level analysis revealed that attachment avoidance had a significant effect on DT bias (**Table 2**); however, the interaction between attachment avoidance and facial expressions was not significant.

**Table 2:** Summary of a 2-level random intercepts liner regression.

Fixed effects		DT			FC	
		
	Estimate	*SE*	*t*	Estimate	*SE*	*t*
Neutral	641.342	87.901	7.296***	0.689	0.189	3.653***
Sad	-222.000	37.554	-5.912***	-0.733	0.100	-7.305***
Happy	-131.310	32.966	-3.983***	-0.370	0.082	-4.508***
Avoidant	-12.471	4.791	-2.603**	-0.026	0.010	-2.479*
Sad × Avoidant	1.282	2.287	0.561	0.004	0.006	0.594
Happy × Avoidant	1.547	1.898	0.815	0.002	0.004	0.557


*DT, the looking time bias for infant faces; FC, fixation count for infant faces; Estimate, mean scores of estimates relative to baseline (neutral and avoidant, respectively). ^∗^*p* < 0.05, ^∗∗^*p* < 0.01, ^∗∗∗^*p* < 0.001.*

The unconditional model without explanatory variables showed that attachment accounted for 89.5% of the variance in infant attentional bias (i.e., intra-class correlation = 0.895). Adding facial expressions (neutral, sad, happy) improved the model fit [χ^2^(148) = 4381.76, *p* < 0.001; likelihood ratio = 6917.48], as did adding attachment [avoidant; χ^2^(147) = 4219.08, *p* < 0.001; likelihood ratio = 6898.32]. Overall, the results in **Table 2** indicate women with attachment avoidance showed a bias toward the negative infant faces out of all the different types of facial expressions.

#### Fixation Count for Infant Faces

The FC was used as another index of visual attentional bias, and the scores were analyzed in the same way as described above. The unconditional model without explanatory variables showed that attachment accounted for 83.6% of the variance in infant attentional bias (i.e., intra-class correlation = 0.836). Adding facial expressions (neutral, sad, happy) significantly improved model fit [χ^2^(148) = 2881.46, *p* < 0.001; likelihood ratio = 1537.68]. Adding attachment (avoidant) also significantly improved model fit [χ^2^(147) = 2770.32, *p* < 0.001; likelihood ratio = 1554.79]. Thus, the results of **Table 2** show that women with attachment avoidance were less likely to fixate on the infant face when paired with an adult face for any of the three types of facial expressions.

## Discussion

In this study, our primary aim was to examine whether adult attachment could be related to infant preference based on visual attention. Specifically, we tested whether nulliparous women would show sustained overt visual attentional biases to infant faces with different facial expressions (neutral, sad, and happy), and whether such biases were influenced by individual differences in adult attachment. The results showed that adult attachment significantly modulated visual attentional bias to infant and adult faces. Multilevel regression analyses indicated that women with higher attachment avoidance had less attentional bias for infant faces compared with adult faces for all three facial expressions, based on total viewing time and total fixations. These results are consistent with our hypothesis that attachment avoidance is related to infant preference, as measured with eye movement indices.

From a facial expressions-level analysis, our results showed that higher DT (longer looks) and FC (more looks) scores, which are both widely used eye indicators depicting attentional bias, were related to infant faces compared with adult faces for all three facial expressions. This verifies that infant faces displaying infant schema described by Lorenz (1943) captures attention (Brosch et al., 2007). Furthermore, the happy and sad expressions scored less than the neutral expressions did. This result indicates that various facial expressions might affect attentional bias, especially neutral expression, which might weaken the baby schema effects. From an eye-movement indices perspective, this finding is consistent with the results of Ding et al. (2016), and further supports the speculation that neutral faces, as an ambiguous expression, are more complex in cognitive processing, while infant faces, as hedonic stimuli, have unique biological significance.

Based on the individual-level analysis, our results showed that higher scores in attachment avoidance had less DT and FC bias for infant faces compared with adult faces for all three facial expressions. This finding indicated that college women with higher attachment avoidance had less sustained overt visual preference for infant faces. From an eye-movement indices perspective, this was in line with findings reported by previous studies indicating that avoidant women are less interested in having children and anticipate less satisfaction from caring for infants (Rholes et al., 1997; Rholes et al., 2006), and was associated with imagining prospective children as less secure and less affectionate (Kwako et al., 2010; Trillingsgaard et al., 2011).

An explanation for this finding may be that the link of two interactional behavioral systems—attachment and caregiving—is at work in attachment theory (Bowlby, 1969). Increased activation of the parents’ attachment system may reduce their own caregiving system activation, thus interfering with childcaring. Under conditions of threat, even adults are likely to be focused on their need for protection; therefore, they lack the mental resources necessary to attend empathically to others’ needs and engage in caring behavior. Following this view, researchers argued that attachment insecurity could create difficulties in attending to others’ distress and in providing care (Feeney and Collins, 2001; Mikulincer and Shaver, 2005). Therefore, since this study’s results indicate that college women with higher attachment avoidance show attentional avoidance for infant faces, they might keep away from infants, who are vulnerable individuals with unstable emotions and the need for intense care.

In addition, preferences for infant faces can be considered a universal explicit representation of parenting motivation (Glocker et al., 2009) and the neuroendocrine system modulate can be seen as the link between parenting motivation and attention (Swain et al., 2011, 2014). Thus, we infer that avoidant women may communicate less with infants with their eyes. The results that women with higher attachment avoidance had less attentional bias for infant faces, according to the eye movement indices perspective, verify our deduction and provide evidence for avoidant women’s lack of preference for infant faces.

In the early days of parenting, parents try to make direct eye-to-eye contact within the newborn’s visual field as much as possible (Simpson, 2001). This helps the infant more easily recognize the parent as a person, thus enhancing the quality of the mother–infant relationship. One previous study indicated that greater attentional bias to infant distress cues was correlated with better scores on a parental bonding questionnaire (Pearson et al., 2010). As far as neurobiology is concerned, benign interactions with the infant may help maintain positive parental attentiveness based on parental oxytocin and dopamine levels (Strathearn et al., 2009; Macdonald et al., 2013). By contrast, if parents’ interactions with their infants are stressful and aggravating, the reward-motivation pathways are less activated; thus, parents are less willing to maintain such interactions (Swain et al., 2014). The most recent eye-tracking study demonstrated that individuals with attachment avoidance were more likely to avoid overtly attending to facial emotional (i.e., angry and happy) stimuli (Byrow et al., 2016). Therefore, our present research results may suggest that the eye movement bias of highly avoidant individuals translates into a lack of eye contact when communicating with their infant child in real situations. This may, in turn, impair the parent–child relationship.

Despite the fact that nulliparous adults have merely the buds of parenting motivation (Scharf and Mayseless, 2011), it has been evolutionarily ingrained into their brains (Swain et al., 2011; Jones et al., 2015). According to attachment theory (Bowlby, 1969), parenting buds reflect a caregiving motivational system that is genetically determined in mammals, which means that they are born with the capacity to perform the caregiving behaviors their offspring need (Mikulincer and Shaver, 2007). In other words, adults can begin thinking about the possibility of being parents before they actually make that decision. This suggests the existence of a universal neurological “caregiving instinct” (Young et al., 2016). Accordingly, parenting may be relatively consistent throughout a variety of life stages, from nulliparous college women to mothers.

Although our results contribute to a better understanding of the relationship between adult attachment and infant preference, our study has several limitations that should be improved in future research. First, our participants were all college women who had never had any children. Brain activation in non-parents differs from that of parents with regard to the right amygdala-induced negative emotions, especially fear and sadness, when they view an infant crying or laughing (Seifritz et al., 2003). Given attachment dynamics across the lifespan (Jchopik et al., 2013), future research should take into account different stages of life in relation to parenthood (e.g., being in love, going through pregnancy, and having children). Second, eye movement indices only provide indirect evidence for maternal behaviors. Further work is needed to investigate the direct effect of this attentional bias on actual caregiving, incorporating both physiological and behavioral indices. Third, in this study we only used facial images, which is only one type of stimulus useful for assessing participants’ infant preference. Alternative experimental stimuli, such as auditory baby cues, should be considered in future studies (Lorberbaum et al., 2002).

Notwithstanding these shortcomings, the results of this study showed that adult attachment orientations might be related to infant preference in terms of explicit behavioral response (i.e., eye movement indices). In addition, they suggest that women with higher attachment avoidance may lack attentional preference for infant faces. These findings have potential significance in their treatment applications and remediation for at-risk mothers and mothers with insecure attachment (e.g., how to predict the mother’s maltreatment of her infant and how to intervene). Such interventions may help improve the quality of the parent–child relationship for highly avoidant women.

## Author Contributions

All authors listed, have made substantial, direct and intellectual contribution to the work, and approved it for publication.

## Conflict of Interest Statement

The authors declare that the research was conducted in the absence of any commercial or financial relationships that could be construed as a potential conflict of interest.
